# Bacteria-Based Roles in Solid Tumors: Potential for Prevention and Treatment

**DOI:** 10.3390/pathogens14090874

**Published:** 2025-09-02

**Authors:** Jianchang Huang, Ailin Zhang, Jialin Sun, Yuhan Fu, Weinan Li, Yanhong Wang

**Affiliations:** 1 School of Pharmacy, Heilongjiang University of Chinese Medicine, Harbin 150040, China; 18845750231@163.com (J.H.); 15504613250@163.com (A.Z.); amelia279@163.com (Y.F.); 2 Key Laboratory of Basic and Application Research of Beiyao, Heilongjiang University of Chinese Medicine, Ministry of Education, Harbin 150040, China; 3 Department of Medicine, Heilongjiang Minzu College, Harbin 150066, China; klp15sjl@nefu.edu.cn

**Keywords:** bacteria, tumors, prevention, treatment

## Abstract

Malignant tumors have become one of the most important diseases threatening human life and health, and the prevention and treatment of cancer have always been the direction of modern medicine’s continuous exploration. According to modern medical research, a tumor microbial ecosystem exists in all human cancers. With the continuous deepening of research on the tumor microenvironment (TME), it has been discovered that some specific bacteria cause tumor production and development by damaging DNA, activating oncogenic signaling pathways, suppressing anti-tumor immunity, and producing pro-tumor metabolites. Certain bacteria associated with tumors can also serve as breakthroughs in the prevention and treatment of tumors. In this review, we present and summarize evidence from a large number of studies on the effects of oncobacteria on tumor prevention and treatment, and we further discuss the direction in which oncobacteria can be positively and effectively used in tumor therapy.

## 1. Introduction

With the continuous progress of modern medicine, more and more diseases have developed mature treatment protocols, and human health has been improved as never before. However, along with the rapid social and economic development of the world, the aging of the population has become increasingly serious, and the global cancer incidence and mortality rates have been climbing. Some studies have shown that it is expected that by 2050, there will be 35 million cases of cancer globally, a 77% increase from 20 million cases in 2022 [1]. Malignant tumors, originating from abnormal cell proliferation, have the characteristics of invasion and metastasis. Nowadays, although early resection of tumors and postoperative chemotherapy and radiotherapy have certain therapeutic effects, they also come with toxicity and adverse reactions [2]. Consequently, investigating novel treatments for tumors is crucial. As a new hotspot of tumor therapy research nowadays, it has been gradually recognized that tumor-associated bacteria and their metabolites play a key role in tumor pathology [3,4]. They not only promote or inhibit tumorigenesis and progression but are also closely related to tumor metastasis. Thus, tumor-associated bacteria are emerging as new therapeutic targets for tumor therapy. In this paper, we begin by discussing the composition of the tumor microenvironment (TME) to describe its characteristics in detail. We take colorectal, breast, and pancreatic cancers as examples to analyze the intrinsic and extrinsic connections between bacteria and tumors, summarizing the mechanisms by which tumor-associated bacteria can be used to treat tumors from three different perspectives. Finally, the current research and application of bacteria as tumor markers are described, providing a comprehensive and systematic overview of their potential in treating and preventing tumors, as well as their therapeutic benefits.

## 2. Tumor Microenvironment

**Figure 1:**
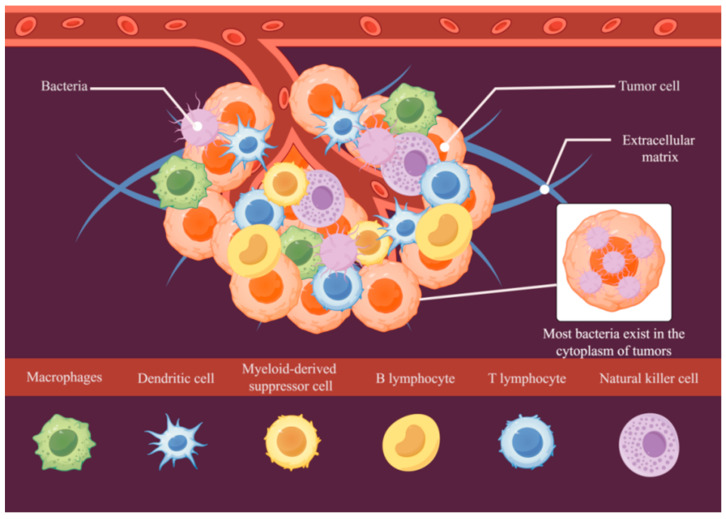
Schematic diagram of the TME.

The TME denotes the biological milieu encompassing malignant cells, comprising two fundamental constituents: noncellular structural components (extracellular matrix) and intratumoral microbial populations (as shown in Figure 1). The extracellular matrix (ECM) in the TME is a complex three-dimensional network structure composed of proteins, polysaccharides, and a variety of molecules. This structural network occupies the intercellular space adjacent to neoplastic cells, offering crucial mechanical support and architectural organization. Its presence is indispensable for preserving tissue homeostasis and structural continuity. Moreover, the ECM is crucial in controlling biological processes, such as cell metastasis and invasion, and it significantly influences tumor growth and dissemination [5]. Among the tumor microorganisms are bacteria, fungi, viruses, and mycoplasma [6]. Of course, tumor cells and immune cells are essential components of the TME. Amongst these, tumor cells play a central role, characterized by an abnormal proliferation rate and invasive ability. They possess a series of unique biological properties, such as the ability to promote angiogenesis through the secretion of a variety of biologically active factors, facilitate the distal metastasis of tumor cells, evade immune surveillance, induce immunosuppression, and inhibit apoptosis [7]. Various types of immune cells exist in the TME, where they function as immune monitors and anti-tumor agents. These cells can recognize and kill abnormal cells, inhibit tumor development, and generate immune memory [8].

There is a special interdependent and symbiotic relationship between tumor bacteria and tumor cells in the TME. Bacteria are capable of entering the cytoplasm of eukaryotic cells, initiating cellular immunity and other cellular responses [9]. The same is true for tumor bacteria, which play different roles in tumorigenesis, growth, and metastasis. During metastasis, for example, they act as facilitators. Tumor cells containing bacteria exhibit stronger adhesion and are larger in size compared to those without bacteria. This suggests a change in the cytoskeleton and attachment capacity. Additionally, this change indicates that bacteria may play a role in the organization of the actin cytoskeleton [10]. Within colorectal cancer (CRC), *Fusobacterium nucleatum* (*Fn*) orchestrates myeloid cell infiltration at infection loci and modulates transcriptional programming in epithelial cells, consequently enhancing local tissue invasion capabilities [11]. In studies on spatial transcriptomics, it has been shown that microbial communities in tumors are not randomly distributed but exist highly organized in specific microecological niches and often play a role in promoting tumor progression [11,12]. Evidence demonstrates that tumor-associated bacteria primarily reside inside cancer cells’ cytoplasm, while their presence in surrounding tissue spaces is comparatively limited [10,13]. Compared with bacteria in the TME, intracellular bacteria can directly enhance tumor cell metastasis ability by reshaping the cytoskeleton [14]. Moreover, taking *Salmonella* as an example, intracellular bacteria often induce DNA damage through ROS. Bacteria in the TME, such as *Escherichia coli*, tend to break DNA double strands by secreting toxins [15]. Overall, the TME contains a large number of bacteria, which are often inextricably linked to tumor development, growth, and metastasis.

## 3. Relationship Between Bacteria and Tumors

Studies targeting genomic aberrations and dysregulated signaling pathways have long been viewed as key to tumor research [16]. However, with the development of culture and identification techniques, when it was discovered that *Helicobacter pylori* in gastric ulcers can induce gastric cancer, the view of the relationship between tumor-bearing bacteria and tumorigenesis changed [17]. More attention has been paid to the relevant effects of tumor microorganisms, especially tumor bacteria, on tumor lesions, growth, and metastasis, and their role in tumor control [18]. With the development of next-generation sequencing (NGS) technology, it is possible to make clearer observations of microbe-rich samples. At the same time, this has brought new opportunities for the study of tumor microbes, e.g., after sequencing of fecal samples from different stages of CRCs and analyzing the sequencing of CRC tumors, it was found that some specific microbes may be present in tumors and their microenvironments [3,11,19], such as *Fn* enrichment in CRC [20,21]. Recent years have seen extensive research into the interactions between microorganisms and malignant tumors, with studies increasingly revealing the critical biological roles of specific microbial communities in tumorigenesis. For instance, *Helicobacter pylori* has been widely documented in clinical studies as a pathogen not only involved in initiating gastric mucosal lesions but also driving the pathological progression of gastric cancer through molecular mechanisms such as regulating the nuclear factor kappa-B (NF-κB) signaling pathway and inducing chronic inflammatory responses [22,23,24,25]. Indeed, bacteria can directly manipulate host cells, altering their normal physiology and enhancing their carcinogenicity [26]. In addition, bacteria can induce the development of cancerous lesions through the induction of chronic infections, immune escape, and immunosuppression [27,28].

From the perspective of inducing chronic infection, bacteria usually interfere with the normal course of the cell cycle through prolonged infections. This interference may lead to changes in cell growth patterns, which, in turn, can trigger DNA damage that is similar to the effects caused by oncogenes [29]. Additionally, chronic infections caused by bacteria can stimulate an immune response in the body, a response that exacerbates oxidative stress in infected cells. During this process, there is an increase in the production of reactive oxygen species (ROS), which are capable of disrupting the integrity of cell membranes and causing DNA damage, leading to cell membrane and DNA damage [30,31]. Not only that, Yang et al. found that gut bacteria use lipopolysaccharide as a trigger to regulate monocyte-like macrophage accumulation in a chemokine-dependent manner and generate a precancerous inflammatory milieu to facilitate tumorigenesis [32].

From the perspective of bacterial toxins and metabolites, some intestinal bacteria are capable of producing *β*-glucuronidase, which triggers the uncoupling of splicing toxins and bile acids, thereby increasing the likelihood of carcinogenesis [33,34,35]. Additionally, bacteria may interfere with normal cellular transformation processes, secrete harmful toxins, disturb the physiological homeostasis of the host, and promote the abnormal proliferation of epithelial and immune cells, all in ways that contribute to the formation of tumors [36].

**Figure 2:**
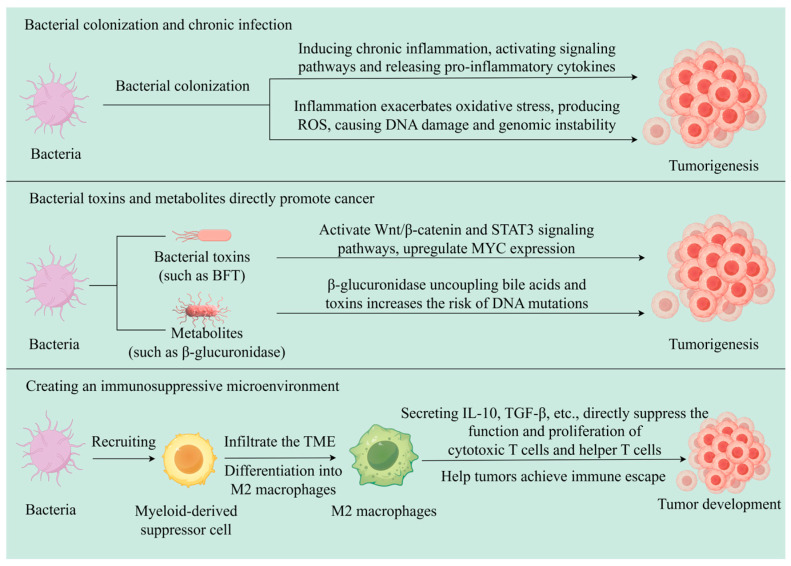
Schematic diagram of the bacteria–TME interaction.

The immunomodulatory and immune-evasion effects of bacteria on tumors often arise from chronic inflammatory environments induced by bacteria, as well as bacterial toxins and their metabolites. For example, enterotoxigenic *Bacteroides fragilis* (ETBF) produces *Bacteroides fragilis* toxin (BFT), which directly acts on intestinal epithelial cells, activating the Wnt-β-catenin pathway, upregulating MYC expression, and triggering NF-κB signaling. This cascade induces chronic inflammation and drives colorectal carcinogenesis [37,38,39,40]. Wu et al. discovered that in multiple intestinal neoplasia (MIN) mice, ETBF induces the production of signal transducer and activator of transcription-3 (STAT3) in epithelial cells, thereby activating a selective T helper 17 cell response. This immune response subsequently recruits CD4+ T cell receptor-αβ (TCRαβ)^+^ and CD4^−^CD8^−^ TCRγδ^+^ T cells, collectively generating a pro-inflammatory TME that drives colorectal carcinogenesis [41]. In a CRC mouse model, Chiu et al. found that Fn has effects on increasing tumor burden, promoting inflammation, and enhancing the infiltration of CD11b+ myeloid-derived suppressor cells (MDSCs) [42]. MDSCs are precursors of tumor-associated macrophages (M2 macrophages), granulocytes, and dendritic cells, and they play an important role in promoting tumor development. Kostic et al. found that the number of M2 macrophages significantly increased in the TME of CRC mice fed Fn, while CD4+ T cells were inhibited [43] (as shown in Figure 2).

Studies have shown that each human cell carries at least 10 bacteria, and this large number of bacteria has different roles in the production and growth of various tumors [44]. However, identifying tumor-associated bacteria and their mechanisms of action is an extremely challenging task. For example, more than 500 bacterial species inhabit the colonic lumen as the resident microbiota [45], and it is undoubtedly very difficult to precisely determine which specific bacteria are likely to induce which specific tumor. In addition, the long latency period between bacterial infection and tumor development makes it extremely difficult to trace the bacterial cause of a tumor back to its source. Correspondingly, an enhanced understanding of bacterial roles in specific malignancies will provide novel avenues for cancer research and therapeutic exploration. Below, we will use three common and representative types of cancer as examples to illustrate the relationship between bacteria and tumors.

### 3.1. Colorectal Cancer and Bacteria

CRC ranks among the most prevalent cancers globally, accounting for substantial mortality in both developed and developing nations [46]. Multiple factors contribute to the development of CRC. Among these, ecological dysregulation within the TME, manifested as an imbalance in the tumor microbiota (particularly the bacteria), plays a crucial role in the development of CRC [47]. Comparative analyses demonstrate elevated *Fn* levels in both neoplastic tissues and fecal specimens from CRC patients relative to healthy controls. Experimental evidence from CRC murine models further indicates that *Fn* enrichment enhances intratumoral heterogeneity while mediating selective recruitment of tumor-associated myeloid populations, with subsequent modulation of immune cell infiltration dynamics. *Fn* generates a pro-inflammatory microenvironment that facilitates tumor progression. In addition to ecological dysregulation, damage to the mucosal barrier is also a cause of CRC [48]. A mucosal barrier exists in the colon, and bacteria can build up a biofilm, which, in turn, destroys the mucosal barrier, triggering chronic mucosal inflammation, prompting an increase in the level of pro-inflammatory cytokines, and increasing the incidence of CRC [49]. Not only that, but bacteria can also induce CRC lesions by damaging host DNA. For example, *Escherichia coli* (*E. coli*) expressing polyketide synthase can alkylate DNA and produce corresponding DNA adducts, damaging the DNA of colonic epithelial cells and thereby inducing CRC [50]. In addition to their role in inducing CRC, bacteria have also been implicated in promoting CRC. In a study, it was found that CRC mice had a shortened survival time and increased mortality when co-colonized with *Bacteroides fragilis* and *E. coli* expressing polyketide synthase [51].

Bacteria not only play roles in the initiation and progression of CRC but also influence tumor therapy. Studies have demonstrated that *Fn* can activate the autophagy pathway in CRC by regulating *microRNA* expression levels, and the activation of this autophagy pathway contributes to enhanced chemotherapeutic drug resistance in tumor cells [52,53]. *Akkermansia muciniphila* reduces colon cancer by activating cytotoxic T-lymphocytes in mesenteric lymph nodes and inducing production of tumor necrosis factor-α (TNF-α) [54]. These studies conclusively demonstrate that tumor-associated microbiota, particularly tumor bacteria, play crucial roles in the initiation, progression, and therapeutic management of CRC.

### 3.2. Breast Cancer and Bacteria

Current epidemiological data indicate that breast cancer has emerged as the most prevalent cancer in female populations, superseding lung cancer in global incidence rates and representing a significant women’s health challenge. The mammary gland has long been considered a sterile organ. However, breast milk samples from lactating women have been found to harbor diverse bacterial communities, and breast tissue itself contains a significant number of distinct bacterial species [55]. Microflora dysbiosis is prevalent in breast cancer tissues as compared to normal breast and is also found to be significantly higher in paracancerous tissues than in healthy controls, particularly regarding the abundance of *Bacillus*, *Staphylococcus*, and *Fn*, which are significantly higher than in normal breast or benign breast lesions [56]. This observation raises the question of whether the bacterial communities in breast cancer might be present before cancer develops or whether the flora in the paraneoplastic tissue promotes breast cancer.

Breast cancer metastasis is a leading cause of patient mortality. It has been found that microbial biomass within breast tumors is low, and its survival benefit to tumor cells is mainly observed during metastasis. Mechanistic studies have shown that during tumor metastasis, circulating tumor cells harboring bacteria enhance their resistance to fluid shear stress through actin cytoskeleton remodeling, thereby facilitating cancer cell metastasis [10]. Some experiments have demonstrated a significant reduction in breast cancer lung metastases after removal of bacteria from the tumor, but primary breast cancer tumor growth was not affected. For example, in a mouse breast cancer model, breast cancer metastatic ability was enhanced in tumor-bearing mice after intratumoral injection of bacteria of human breast cancer origin, and it was inhibited in tumor-bearing mice after elimination of bacteria from the breast cancer using antibiotics [10]. Reduced intratumoral microbial diversity in breast cancer impairs immune cell recruitment and activation within the TME, thereby facilitating tumor growth and metastatic progression [57]. In addition, it has been shown that a decrease in the concentration of the bacterial metabolite lithocholic acid reduces the apoptotic effects of the oxidative stress it induces in cells, and breast cancer may then be able to develop [58]. This shows that changes in metabolites associated with intratumoral bacteria in breast tumors can also cause pathologic changes associated with a tumor.

In a practical study, it was found that in a mouse breast cancer model, Fap-2, the membrane protein of *Fn*, binds to galactose-N-acetylgalactosamine (GalNAc), which is highly expressed in breast cancer cells. This binding allows *Fn* to specifically colonize breast cancer tissues. CD4+ and CD8+ cell levels in AT3 breast tumors of infected and uninfected mice were compared by flow cytometry, and the results showed that *Fn* reduced CD4+ and CD8+ cells and promoted breast cancer growth and metastasis [58]. Furthermore, *Fn* induces DNA damage in breast cells and promotes breast cancer by regulating the bioavailability of estrogen [59]. These studies have fully demonstrated that tumor-associated bacteria have a significant impact on the progression and development of breast cancer lesions, playing an especially important role in breast cancer metastasis.

### 3.3. Pancreatic Cancer and Bacteria

Pancreatic cancer poses a significant threat to human health due to its extremely high mortality rate. More bacteria are present in the pancreas of pancreatic cancer patients compared to the normal pancreas, and they are abundant. In an experimental study, after testing 113 pancreatic samples with pancreatic ductal adenocarcinoma and 20 healthy pancreatic samples, it was found that the bacterial detection rate of the pancreatic ductal adenocarcinoma samples was much higher than that of the healthy pancreatic samples [60]. Emerging evidence reveals that bacterial components exert critical modulatory functions within the pancreatic cancer TME, demonstrating multidimensional regulatory influence on both cancer pathogenesis and therapeutic responses. For example, pancreatic cancer patients with long survival have strong immunomodulatory properties associated with the *Saccharopolyspora* and Gram-positive spore-forming bacilli that are enriched in their tumors [4]. It has been shown that intratumoral bacteria in pancreatic cancer drive the establishment of a tumor immunosuppressive microenvironment through the activation of Toll-like receptors (TLRs) and that these bacteria selectively activate TLRs in monocytes and induce differentiation of M2-like tumor-associated macrophages. Furthermore, it has been demonstrated experimentally that antibiotic clearance of intratumoral bacteria significantly promotes T-cell activation, M1-like tumor-associated macrophage differentiation, and PD-1 protein upregulation while decreasing myeloid-derived suppressor cells and M2-like tumor-associated macrophages within the tumor. Isolation of infiltrating T cells from in situ pancreatic cancer tumors in antibiotic-treated mice and injection into a subcutaneous tumor model of pancreatic cancer in mice resulted in a decrease in tumor weight of about 50% [61]. Tumor-associated bacteria can influence all stages of tumor development, which provides multiple strategies for leveraging bacteria in cancer therapy. A summary of the common intratumoral bacteria in colorectal, breast, and pancreatic cancers is shown in Table 1.

## 4. Bacteria for Tumor Prevention and Treatment

**Figure 3:**
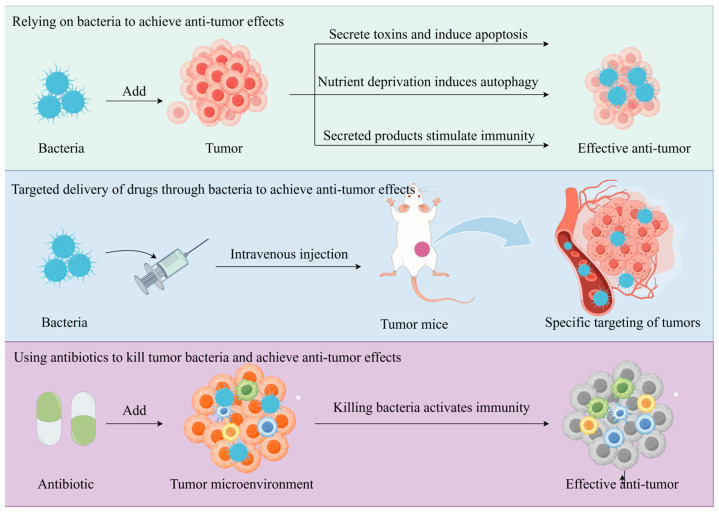
Introductory diagram showing three modes of action of tumor bacteria in tumor control (mode 1 is the anti-tumor effect achieved by relying on the bacteria’s own components or products; mode 2 is the tumor-targeting effect of some specific bacteria; and mode 3 is the anti-tumor effect achieved by relying on the activation of autoimmunity after the destruction of the tumor bacteria by antibiotics).

As documented in the 19th century, tumor regression occurred in cancer patients receiving *Streptococcus pyogenes* and *Serratia marcescens* inoculations as therapeutic interventions [79]. Therefore, bacteria began to be regarded as a potential cancer treatment method, and the use of bacterial components or products to achieve anti-tumor effects proved to be feasible. With continued research on the relationship between bacteria and tumors, bacteria such as *Clostridium difficile*, *Bifidobacterium*, *Salmonella*, *Proteus*, *Lactobacillus*, and *Escherichia* have been shown to preferentially accumulate in a hypoxic, immunosuppressed TME, with the ability to play an immune-stimulating role, with the potential to act as tumor-targeting vectors [80]. According to research, certain bacteria may have the effect of inducing or promoting the growth and metastasis of specific tumors. Accordingly, the use of antibiotics and other methods to kill bacteria in tumors to achieve the effect of tumor treatment has also been confirmed in experiments [81]. Indeed, the promotion or inhibition of tumor growth by tumor bacteria is often combined with a specific tumor environment, and these interactions reflect the complex relationship between tumor bacteria and tumor immunity [82,83]. The roles that bacteria can play in tumor prevention and tumor therapy are described below in three dimensions (as shown in Figure 3).

### 4.1. Antitumor Effects of Bacterial Components or Products

As early as the 19th century, it was found that tumor regression was successfully observed in patients by injecting *Streptococcus pyogenes* and *Serratia marcescens*. From then on, this discovery opened the door to studying the anti-tumor effects of bacteria and promoted the use of bacteria in research aimed at inducing tumor regression and even apoptosis. Through continuous research, it has been found that the proliferation of bacteria in tumors induces the migration of innate immune cells, such as macrophages, neutrophils, and dendritic cells (DCs), to the tumor [84]. Emerging research delineates a plethora of tumor-suppressive pathways inherent in tumor bacteria, revealing their intrinsic capacity for multimodal anti-tumor activity [85,86,87,88]. For example, *Salmonella* can directly kill tumor cells by producing toxins that induce apoptosis and deprive tumor cells of nutrients, causing them to autophagize [89,90,91,92,93]. *Salmonella* demonstrates bimodal anti-tumor activity, combining direct tumoricidal effects with immunomodulatory capacity mediated through Connexin43 (Cx43) overexpression. This bacterial-induced Cx43 elevation facilitates DC-tumor cell gap junction formation, enabling intercellular transfer of tumor antigens that initiates DC-dependent cross-priming of CD8+ T lymphocytes, ultimately orchestrating adaptive immune-mediated tumor clearance [94,95]. These anti-tumor mechanisms were also confirmed experimentally in a mouse model of melanoma injected with attenuated *Salmonella typhimurium*, which showed tumor regression in the injected mouse model compared to a mouse model not injected with Salmonella-specific T cells [96]. Through in vivo and in vitro experiments, a team verified that the use of *Listeria monocytogenes* (*LM*) and Listeriolysin O (LLO), the major virulence factor of *LM*, can effectively inhibit tumor growth and metastasis. LM-LLOLM-LLO activates Nicotinamide Adenine Dinucleotide Phosphate (NADPH) oxidase in macrophages and neutrophils, which, in turn, generates ROS and induces 4T1 and MCF7 tumor cell death. In addition to NADPH oxidase-mediated ROS, LM-LLO increases intracellular Ca^2+^ levels; both approaches contribute to LM-LLO-induced mitochondrial failure in tumor cell death [97]. Similarly, it has been demonstrated that transplantation of long-surviving fecal microorganisms from patients with pancreatic ductal adenocarcinoma (PDAC) into a mouse model of PDAC led to the upregulation of CD8+ T cells to produce tumor immunity, which ultimately succeeded in inhibiting tumor growth in the mice [4].

Not only is there an anti-tumor effect in the tumor bacteria themselves, but some of the components in the tumor bacteria also act as anti-tumor agents. For example, Lipopolysaccharides (LPSs) and flagellin are present in *Salmonella*. They both promote anti-tumor effects. LPSs interact with TLR4 to directly activate macrophages and DCs to produce interleukin-1β [98]. Moreover, LPSs interact with CD14, TLR4, and myeloid cells to elevate tumor necrosis factor-α secretion, which acts as an anti-tumor agent [99,100,101]. Additionally, flagellin works together with TLR5 to downregulate the number of CD4+ and CD25+ regulatory T cells, leading to tumor suppression [102].

The secretion products of tumor bacteria also play a non-negligible role in anti-tumor activity. For example, *Clostridium* difficile infection secretes a variety of toxins that can induce the recruitment of granulocytes and cytotoxic lymphocytes to the TME. These changes can lead to an increase in the levels of various cytokines and chemokines, thereby improving anti-tumor capacity [103,104]. In studies of *Faecalibacterium prausnitzii*, it was discovered that advanced gastric adenocarcinoma patients with high abundance of *Faecalibacterium prausnitzii* exhibited inhibited tumor progression during immune checkpoint inhibitor therapy. This effect occurs because Faecalibacterium prausnitzii produces compounds that inhibit histone deacetylases (HDAC), thereby promoting the secretion of IFN-γ and granzyme B by CD8+ T cells [105].

In addition to this, the use of bacterial engineering can also provide a good anti-tumor effect. Expression of L-asparaginase with *Salmonella typhimurium* disrupts cellular metabolism and effectively inhibits tumor growth [106]. With bacterial engineering, the expression of apoptosis-inducing cytotoxic molecules can also effectively inhibit tumor growth, e.g., secretion of pro-apoptotic TNF family cytokines can selectively induce cytotoxicity to inhibit tumor development [107]. Moreover, since macrophages in the TME are often polarized to the M2 type, exhibiting immunosuppressive effects, the bacterial engineering of *Salmonella typhimurium* to secrete exogenous *Vibrio* traumaticus-derived flagellin B can transform immunosuppressive M2 macrophages into pro-inflammatory M1 macrophages in the TME, activate immune responses, and produce tumor-suppressive effects [108]. In a recent study by Chen et al., a nano-system termed TM@^CD326^hOMV was constructed through bacterial engineering. This system utilizes hybridized bacterial outer membrane vesicles (hOMVs) derived from *Akkermansia muciniphila* and CD326-targeting peptide-engineered *Escherichia coli* as carriers, loaded with the copper chelator tetrathiomolybdate (TM). In breast cancer murine models, this nano-agent depleted copper ions, thereby reversing immunosuppression and increasing infiltration of natural killer (NK) cells and CD8+ T cells by 3-fold. When combined with a PD-1 inhibitor, it significantly extended murine survival [109]. These experimental studies have demonstrated the direct role played by tumor bacteria and their products in tumor therapy.

The traditional chemotherapy cancer treatment program has the defects of drug resistance and non-specific toxicity to normal body cells. In contrast, bacteria-based anti-tumor therapy demonstrates more anti-tumor pathways and shows greater developmental potential. However, relying on bacterial components or products as anti-cancer drugs also has its own limitations. To achieve a certain therapeutic effect, high doses of bacteria are often required, and the resulting innate toxicity and pathogenicity are unavoidable challenges. If the dosage is reduced, it may lead to decreased efficacy, while maintaining a high dosage may result in infection or even death. Therefore, a number of researchers have attempted to overcome this shortcoming by starting with virulence-reducing and genetically modified strains.

### 4.2. Natural Targeting of Bacteria to Tumors

Tumor generation and growth often cause unique pathological changes at the tissue level, and the vascular system surrounding the tumor usually develops irregularly and chaotically, resulting in a hypoxic, acidic environment for most solid tumors [110,111]. They provide ideas for tumor targeting while limiting the effectiveness of chemotherapy. Standard chemotherapeutic agents exhibit limited diffusion capacity within hypoxic tumor microenvironments [112]. This compromised penetration not only diminishes intratumoral drug accumulation but also promotes off-target biodistribution, collectively contributing to therapeutic inefficacy [113] and ultimately leading to therapeutic failure. Therefore, whether it is possible to combine the hypoxic and acidic TME characteristics with tumor-targeted drug release opens a new way of thinking about tumor targeting. In the past, nanoparticles, biological camouflage, or magnetic induction were often utilized to deliver chemotherapeutic agents [114,115]. However, both micellar nanoparticles and targeted nanoparticles, designed by attaching biorecognition molecules to the surface of nanoparticles, are subject to further examination in terms of feasibility. Consequently, researchers have begun exploring the potential of developing novel drug delivery systems. Given that anaerobic bacteria can efficiently accumulate in hypoxic tumor regions, they have emerged as a promising new option for tumor-targeted drug delivery platforms. The strategy of targeting tumor tissues through bacteria differs from the passive diffusion and accumulation of traditional drugs, which tend to accumulate only in the vicinity of blood vessels. Bacteria can actively penetrate into the depths of the tumor to better achieve the targeting effect [116,117].

It has been found that the number of bacteria that reach a target tumor tends to be roughly the same as the number of bacteria that reach other normal tissues [118,119,120]. But, whereas in other healthy tissues, bacteria are cleared within days, in the tumor environment, some specific bacteria colonize and proliferate [121,122]. For example, *Salmonella* will preferentially enter and colonize the TME [123], and the ratio of *Salmonella choleraesuis* colonizing tumor sites to non-tumor sites exceeds 1000:1 [124]. In fact, the reason why bacteria can colonize and proliferate in tumors is also related to the fact that bacteria change the TME during the process of targeting and colonizing tumors. The presence of bacteria in tumors stimulates the host’s own immune response, leading to an influx of immune cells, including neutrophils, into the tumor area. These neutrophils gather in large numbers and are scattered around the necrotic regions of tumors, surrounding the bacteria present there. When neutrophils encounter bacteria, they release a type of neutrophil extracellular trap composed of antibacterial proteins and chromatin fibers, which limits the range of bacterial activities in the tumor and, at the same time, provides favorable conditions for bacterial proliferation [125,126]. This also explains why facultative anaerobes, which would otherwise be distributed throughout a tumor, tend to concentrate only in necrotic areas of a tumor.

**Figure 4:**
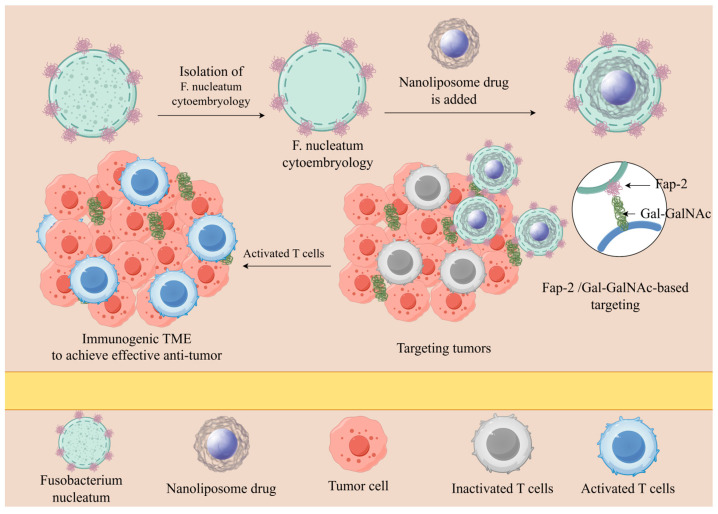
A diagram of the anti-tumor nanomedicine targeting delivery mechanisms that rely on bacterial targeting. (Tumor-targeted delivery of drugs is achieved by isolating *Fn* cell membranes and adding liposomal nanodrugs to form nanodrugs that are coated with *Fn* cell membranes, based on the targeting relationship that exists between the membrane protein Fap-2 on *Fn* cell membranes and the tumor marker Gal-GalNAc).

Furthermore, in studies of obligate anaerobes and facultative anaerobes, it was found that anaerobes do not colonize hypoxic environments unrelated to tumors [87,122,127]. More and more studies have found that bacteria such as *Listeria*, *Salmonella*, *E coli*, and *Fn* can be actively targeted to hypoxic tumor regions, and the protocol of using anaerobic bacteria as a tumor-targeting substrate has been adopted and researched in a large number of cases [116,128,129]. (A diagram of anti-tumor nanomedicine delivery mechanisms that rely on bacterial targeting is shown in Figure 4).

A research team combined the p53 protein, which can directly induce apoptosis in tumor cells, with the Tum5 protein, which has anti-angiogenic function, and constructed a Tum5-p53 bifunctional fusion protein. They then used *E. coli* Nissle 1917 as a targeting vector to deliver it to the hypoxic region of the tumor. The results showed that the engineered *E. coli* exhibited obvious tumor-targeting and inhibitory capabilities [130]. Using attenuated *Salmonella typhimurium* as a targeted vector to express cytolysin A has also shown significant targeting and inhibitory effects in tumor-bearing mouse treatment experiments [131].

In recent years, molecular targeted therapies have shown remarkable therapeutic efficacy, but they also have their own limitations. These targeted therapies are often effective only for tumors with specific gene mutations and have certain off-target effects. In contrast, tumor-targeted therapies based on tumor bacteria have demonstrated greater adaptability and targeting ability. In addition, oncobacteria can be chemically or genetically modified to synthesize anti-tumor drugs and localize them within tumor tissues, which is a safer and more effective process that reduces cytotoxicity to normal cells. With ongoing exploration of bacterial tumor-targeting capabilities, the precision and efficacy of bacterial targeting have been consistently validated, demonstrating remarkable performance that expands therapeutic options for tumor-targeted interventions.

### 4.3. Treatment of Tumors by Destroying Bacteria

Current medical research on tumors has moved beyond the view that tumors are triggered only by genetic or epigenetic changes in cells to the recognition that tumor development is a complex physiological process influenced by a variety of factors, among which bacteria are undoubtedly some of the key contributors. Bacteria can promote the occurrence and development of tumors, so treating bacteria inside tumors has undoubtedly become a potential therapeutic target for oncology treatment. For example, treatment of mice carrying *Fn*-positive patient-derived CRC xenografts with the antibiotic metronidazole effectively killed *Fn* and inhibited tumor growth, resulting in a reduction in tumor size [64]. This suggests that the targeted killing of bacteria inside tumors can serve to treat tumors.

However, targeting intratumoral bacteria and eradicating them remains challenging, especially in ensuring that antibiotics reach effective concentrations in the TME while avoiding their adverse effects on the host. Achieving minimum inhibitory concentrations of antibiotics in the TME is often difficult due to factors such as physical barriers in tumor tissue, inadequate blood supply, and reduced chemical stability of antibiotics in the TME [132]. However, high doses of antibiotics can disrupt the microbial homeostasis of the patient’s body, making it particularly important to develop methods that can accurately target bacteria in tumors with antimicrobial resistance. For example, one team coupled the antibiotics polymyxin B sulfate and sushi peptide with gold nanoparticles to target *Salmonella typhimurium* in cervical cancer tumors, effectively killing the target bacteria. In addition, their constructed nanoparticles showed stronger antimicrobial resistance compared to polymyxin B sulfate alone or sushi peptide alone [133]. This sets the stage for the use of antibiotics at relatively low doses to achieve bacterial killing. There is also a fairly well-established experimental demonstration of protocols aimed at eliminating tumor bacteria in order to treat tumors. In the treatment of CRC, an expanding volume of research demonstrates that the abundance and composition of gut microbiota profoundly impact therapeutic outcomes in CRC patients. Wang et al.’s experiments demonstrated that Fn preferentially accumulates in hypoxic tumors. They also found that in a hypoxic environment, relative to a normal environment, Fn can increase the invasive ability of CRC cells by 15-fold. Moreover, they produced an antibiotic silver–tinidazole complex encapsulated in liposomes. This complex successfully reduced the abundance of primary and metastatic CRC anaerobes without a significant impact on the overall intestinal microbiota. It also successfully activated the targeted immune response to CRC. This had a positive effect on CRC treatment, effectively inhibiting and even eliminating CRC cells [81].

The elimination of tumor bacteria can be achieved not only by antibiotics but also by using phages to eliminate tumor-associated bacteria. For example, *Fn*-targeting phages in combination with irinotecan can selectively inhibit CRC-associated *Fn* in vivo, thereby inhibiting tumor growth [134]. Similarly, direct elimination of adherent invasive *E. coli* by phages also reduces tumor load in a mouse model of CRC [135].

Compared to common anticancer drugs, antibiotic drugs are more readily available and can effectively reduce treatment costs. However, the strategy of using antibiotics to eliminate bacteria in a tumor to achieve anti-tumor purposes also has limitations. For example, there are limitations such as bacterial resistance and difficulty in controlling antimicrobial concentration within tumors. To address these limitations, a combination of tumor-targeted delivery is required. From the three different programs, it is evident that treating tumors by relying solely on a single approach is often insufficient to achieve a complete cure. If we rely only on bacterial treatment of tumors, there will often be a situation where the tumor is not completely eliminated. Therefore, combining bacterial therapy with traditional therapy may be a better choice.

## 5. Bacteria as Tumor Markers

Tumor markers are a class of substances that can reflect the existence and changes in tumors during the occurrence, development, and treatment of malignant tumors. They are often associated with tumor cells and their metabolites. Existing tumor markers include, for example, carcinoembryonic antigen (CEA), carbohydrate antigen 125 (CA125), carbohydrate antigen 19-9 (CA19-9), and so on [136,137]. With the continuous research on tumor therapy, bacteria are emerging as a significant field of tumor marker research. Derosa et al. found that the relative abundance of the mucinophilic protein *Akkermansia muciniphila* (*AKK*) could be used as a biomarker for prognosis in patients undergoing PD-1 blockade immunotherapy [138]. In a similar vein, Gou et al. used Illumina MiSeq sequencing to compare fecal samples of CRC patients and healthy populations. They showed that the ratio of *Fn* to *Faecalibacterium prausnitzii* (*Fp*) (*Fn*/*Fp*) was significantly higher in CRC patients than in healthy population controls, and the ratio of *Fn* to *Bifidobacterium* (*Bb*) (*Fn*/*Bb*) was also significantly higher than in healthy population controls. These studies demonstrate the potential of bacterial ratios in tumor markers [139].

In addition to conventional bacteria serving as tumor markers, genetically engineered bacteria can also be utilized for the precise detection of various malignant tumor types. Peter et al. found that based on the expression of endogenous *E. coli* thymidine kinase, *E. coli* Nissle 1917 (EcN) can be imaged by Positron emission tomography (PET). The tumor-targeting properties of EcN enable it to aggregate in tumors, thus allowing the imaging of solid tumors in oncology patients. This imaging modality also provides a new method for detecting solid tumors [140]. Research on using bacteria as tumor markers has made some progress, but there are still considerable limitations in practical applications. The primary challenge stems from the lack of scientifically unified standards in sample collection methods and data analysis and processing. Microbial databases are mainly obtained through 16S rRNA sequencing and shotgun metagenomics, but there is no clear standard for these techniques in sampling and data analysis. Furthermore, the microbial biomass of many tumor-associated ecological niches is usually relatively low, which adds to the complexity and difficulty of the analysis [41]. Meanwhile, the heterogeneity in the intratumoral microbiota, such as the existence of heterogeneity in the composition of intratumoral microorganisms in different patients, is manifested in the differences in species and abundances of the intratumoral microbiota in different patients. In a related study, 16S rRNA gene sequencing of 44 tumor tissues from 11 CRC patients found that intratumoral microorganisms in the tissues of most of the patients showed different degrees of heterogeneity [14]. Therefore, using the negative–positive bacterial test alone to determine whether a tumor exists in a patient can lead to false-positive results, making it difficult to establish a uniform standard for bacterial markers.

Because of the heterogeneity in the tumor microbiota and the difficulty in establishing uniform standards, the research direction of future bacterial markers may focus more on the development of personalized treatment strategies. The bacterial therapy mentioned earlier utilizes tumor bacteria to achieve therapeutic effects. It is also possible to study corresponding bacterial markers to monitor changes in the number of bacteria at different stages of treatment, thereby providing a basis for bacterial therapy.

## 6. Bacterial Therapy-Related Clinical Trials

Bacterial-based therapy has demonstrated a certain level of feasibility in clinical trials. Bacillus Calmette-Guérin (BCG) is one of the earliest bacteria applied in cancer treatment. In a recent clinical trial (NCT04165317) targeting non-muscle-invasive bladder cancer (NMIBC), tumor shrinkage was observed in some patients following treatment [141]. Luke et al. (NCT04167137) conducted a phase I study on the treatment of advanced malignant tumors. The enrolled participants with refractory advanced cancers received repeated intratumoral injections of SYNB1891 either alone or in combination with atezolizumab. SYNB1891 is a live, modified strain of the probiotic *Escherichia coli* Nissle 1917 (EcN) engineered to produce cyclic dinucleotides under hypoxia, leading to STimulator of INterferon Genes (STING) activation in phagocytic antigen-presenting cells in tumors and the activation of complementary innate immune pathways. In their study, they demonstrated the clinical feasibility of this bacterial therapy [142]. *Clostridium novyi*-NT is a genetically engineered anaerobic bacterium capable of proliferating in the hypoxic regions of tumors. In a clinical trial involving patients with advanced solid tumors, Janku et al. observed significant tumor shrinkage in certain patients following bacterial treatment, confirming both the safety and efficacy of this bacterial therapy (NCT01924689) [143]. In another clinical trial, the attenuated *Salmonella* strain VNP20009 was confirmed to have certain tumor-targeting and anti-tumor effects (NCT00004988).

## 7. Limitations of Bacterial Therapy

Despite its advancing maturity, bacterial therapy still exhibits inherent limitations. The first and foremost among these is the intricate complexity of the bacterium–tumor relationship. In recent years, considerable clinical and experimental data have demonstrated the inextricable links between tumor bacteria and tumors, but these links are complicated by the fact that the development of a single cancer is often associated with a variety of tumor bacteria, and a single tumor bacterium can play multiple roles in a certain cancer type. It is this complex association between bacteria and tumors that makes it difficult to identify the specific bacteria that cause a particular tumor. Moreover, the molecular mechanisms by which bacteria (especially specific subgroups) affect tumor signaling pathways, anti-tumor immunity, and therapeutic efficacy remain unclear. Technological bottlenecks also limit progress, as there is a lack of high-resolution tools (such as single-cell and spatial omics techniques) to accurately analyze the complex microbial composition and spatial distribution in the TME. Significant tumor heterogeneity leads to huge differences in the composition, abundance, and function of microorganisms in different types of cancer (such as CRC, lung cancer, and breast cancer) and even in the same tumor, making it extremely difficult to develop general strategies. In addition, the impact of intratumoral bacteria on tumors is bidirectional—some bacteria (such as *Fn*) promote cancer progression through specific signaling pathways, while others (such as *Lactobacilli*) may inhibit tumor growth through immune regulation. This complexity requires precise localization and regulation of therapeutic interventions. The insufficient coverage of tumor types in the current research also limits the widespread applicability of this strategy. These challenges in terms of mechanisms, technologies, heterogeneity, and complexity collectively constrain the widespread application of this strategy.

## 8. Conclusions and Future Prospects of Bacterial Therapy

A growing body of research suggests that bacteria have an irreplaceable role in human health and play multiple roles in human health. In the ongoing research on bacteria, it has been found that bacteria can alter the normal physiology of host cells by promoting inflammation, altering cell signaling pathways, enhancing immune evasion, and inducing DNA damage. As an emerging therapeutic strategy, bacterial therapy offers unique advantages, and researchers have achieved a series of notable accomplishments in the field of cancer treatment. The current research outcomes in bacterial-based cancer therapy are primarily reflected in bacterial tumor targeting, bacterium-mediated drug delivery, and bacteria-induced immune responses. With deeper insights into tumor biology and immunology, the potential of bacterial therapy holds even broader promise. The key to future breakthroughs lies in deepening basic research by utilizing animal models and advanced technologies, such as gene editing and multi-omics integration, to elucidate the mechanisms of bacterial tumor immune interactions; developing high-resolution tools to overcome technical bottlenecks; designing safer targeted drugs or engineered strains based on the optimization strategy of bacterial biological characteristics; and exploring effective synergistic combination therapies. In short, if the existing obstacles can be overcome, bacterial therapy is expected to fully unleash its enormous potential for precision cancer treatment, diagnosis, prevention of recurrence, and deep innovation in tumor treatment.

## Figures and Tables

**Table 1:** Common intratumoral bacteria in colorectal, breast, and pancreatic cancers.

Tumor Types	Intratumoral Bacteria	Mechanism of Tumor Occurrence	References
Colorectal cancer	*Fusobacterium nucleatum*	Dysregulation of signaling pathways; Inactivation of tumor suppressor genes; Abnormal epigenetic regulation; Immune escape; Polarization of immune cell phenotypes; Genome damage; Chronic inflammation.	[43,61,62,63,64]
	*Escherichia coli*	Dysregulation of signaling pathways; Abnormal epigenetic regulation; Immune escape; Polarization of immune cell phenotypes; Genome damage; Chronic inflammation.	[51,65,66,67]
	*Phylum Proteobacteria*	Dysregulation of signaling pathways; Abnormal epigenetic regulation; Immune escape; Genome damage; Chronic inflammation.	[68,69]
	*Porphyromonas gingivalis*	Dysregulation of signaling pathways; Inactivation of tumor suppressor genes; Abnormal epigenetic regulation; Immune escape; Polarization of immune cell phenotypes; Genome damage.	[70]
	*Prevotella*	Dysregulation of signaling pathways; Abnormal epigenetic regulation; Immune escape; Polarization of immune cell phenotypes; Direct DNA damage.	[68]
	*Peptostreptococcus*	Dysregulation of signaling pathways; Abnormal epigenetic regulation; Immune escape; Genome damage.	[68]
	*Prevotella intermedia*	Dysregulation of signaling pathways; Abnormal epigenetic regulation; Immune escape; Polarization of immune cell phenotypes; Genome damage.	[64,71]
	*Fusobacterium necrophorum*	Dysregulation of signaling pathways; Immune escape; Polarization of immune cell phenotypes; Direct DNA damage.	[64]
Breast cancer	*Methylobacterium radiotolerans*	Dysregulation of signaling pathways; Abnormal epigenetic regulation; Immune escape; Polarization of immune cell phenotypes; Genome damage.	[72]
	*Fusobacterium nucleatum*	Dysregulation of signaling pathways; Abnormal epigenetic regulation; Immune escape; Polarization of immune cell phenotypes; Genome damage.	[73]
	*Bacteroides fragilis*	Dysregulation of signaling pathways; Abnormal epigenetic regulation; Immune escape; Polarization of immune cell phenotypes; Direct DNA damage.	[74]
	*Escherichia coli*	Dysregulation of signaling pathways; Abnormal epigenetic regulation; Immune escape; Genome damage.	[10]
	*Staphylococcus epidermidis*	Dysregulation of signaling pathways; Abnormal epigenetic regulation; Immune escape; Direct DNA damage.	[10]
Pancreatic cancer	*Fusobacterium nucleatum*	Dysregulation of signaling pathways; Chronic inflammation; Immune escape.	[75]
	*Porphyromonas gingivalis*	Dysregulation of signaling pathways; Immune escape; Polarization of immune cell phenotypes.	[76]
	*Pseudomonadaceae*	Direct DNA damage; Chronic inflammation.	[60]
	*Enterobacteriaceae*	Immunosuppressive cell infiltration; Direct DNA damage.	[60]
	*Proteobacteria*	Immunosuppression; Chronic inflammation; Genome damage.	[60]
	*Helicobacter pylori*	Dysregulation of signaling pathways; Genome damage; Chronic inflammation.	[77]
	*Citrobacter freundii*	Dysregulation of signaling pathways; Chronic inflammation.	[78]
